# Possible electric field induced indirect to direct band gap transition in MoSe_2_

**DOI:** 10.1038/s41598-017-05613-5

**Published:** 2017-07-12

**Authors:** B. S. Kim, W. S. Kyung, J. J. Seo, J. Y. Kwon, J. D. Denlinger, C. Kim, S. R. Park

**Affiliations:** 1 Department of Physics and Astronomy, Seoul National University, Seoul, 08826 Korea; 2 Center for Correlated Electron Systems, Institute for Basic Science, Seoul, 08826 Korea; 3 Department of Physics, Incheon National University, Incheon, 22012 Korea; 4 Advanced Light Source, Lawrence Berkeley National Laboratory, Berkeley, CA 94720 USA; 5 Institute of Physics and Applied Physics, Yonsei University, Seoul, 03722 Korea

## Abstract

Direct band-gap semiconductors play the central role in optoelectronics. In this regard, monolayer (ML) MX_2_ (M = Mo, W; X = S, Se) has drawn increasing attention due to its novel optoelectronic properties stemming from the direct band-gap and valley degeneracy. Unfortunately, the more practically usable bulk and multilayer MX_2_ have indirect-gaps. It is thus highly desired to turn bulk and multilayer MX_2_ into direct band-gap semiconductors by controlling external parameters. Here, we report angle-resolved photoemission spectroscopy (ARPES) results from Rb dosed MoSe_2_ that suggest possibility for electric field induced indirect to direct band-gap transition in bulk MoSe_2_. The Rb concentration dependent data show detailed evolution of the band-gap, approaching a direct band-gap state. As ionized Rb layer on the surface provides a strong electric field perpendicular to the surface within a few surface layers of MoSe_2_, our data suggest that direct band-gap in MoSe_2_ can be achieved if a strong electric field is applied, which is a step towards optoelectronic application of bulk materials.

## Introduction

Whether a semiconductor has a direct or indirect band-gap greatly affects its optical properties; excitons in direct band-gap semiconductors, for example, strongly couple to photons. For such reason, direct band-gap semiconductors can be used for optoelectronic applications such as light emitting and laser diodes^1, 2^. Monolayer 2H-MX_2_ (M = Mo, W; X = S, Se) also have a direct band-gap and have drawn considerable attention due to their novel electronic properties as described by the Massive Dirac-Fermion model^3, 4^. The coupling between the valley degree of freedom and circularly polarized light opened the field of valley Hall effect^5, 6^. Such intriguing physics can be realized only in 1 ML 2H-MX_2_ because multi-layer 2H-MX_2_ has an indirect band gap and thus does not strongly couple to light. Photoluminescence and photo absorption spectroscopy experiments indeed show that photon-exciton coupling is strong only in 1 ML 2H-MX_2_^7–10^.

Making bulk MX_2_ a direct gap material would be highly desirable because the necessity for high quality ML MX_2_ puts severe limitation on the actual application. There were many computational or experimental studies in search of a way to control the band structure and gap size in bulk 2H-MX_2_. It was found that the band gap size of TMDs can be modified by using various methods such as strain^11–17^, chemical doping^18–22^, electric field^12, 23–26^, making heterostructures^12, 27–31^ and using different substrates for thin films^32–35^. However, there has not been any proposal to induce direct band gap in bulk MX_2_.

Alkali metal atoms evaporated on the surface of a sample not only dope electrons to the sample but also generate a strong electric field near surface^36^. Our strategy to the issue of inducing a direct gap is to apply a strong electric field perpendicular to the MoSe_2_ layers by using such alkali metal dosing. Valence band dispersions and conduction band minimum (CBM) were measured by angle-resolved photoemission spectroscopy (ARPES). The indirect gap which is about 0.2 eV smaller than the direct gap in bulk MoSe_2_ is greatly reduced after alkali metal dosing, almost to the point of indirect to direct gap transition. Our observation casts a strong possibility for an indirect to direct gap transition under a strong electric field in MoSe_2_.

## Results

### Indirect to direct band-gap transition under an external electric field

First principles calculations show that electronic band dispersions of bilayer MX_2_ can be greatly modified by applying an electric field perpendicular to the layers^23^. Bilayer MX_2_, originally an indirect band gap semiconductor, could even become a metal under a strong electric field. In that process, the location of valence band maximum (VBM) moves from the Γ- (without an electric field) to K-point (with a moderate electric field). This indicates an indirect to direct band gap transition under an electric field even though it was not explicitly discussed in the work due to mis-location of CBM. We point out that recent ARPES experiment results locate CBM at the K-point in MoSe_2_^4, 32^.

**Figure 1:**
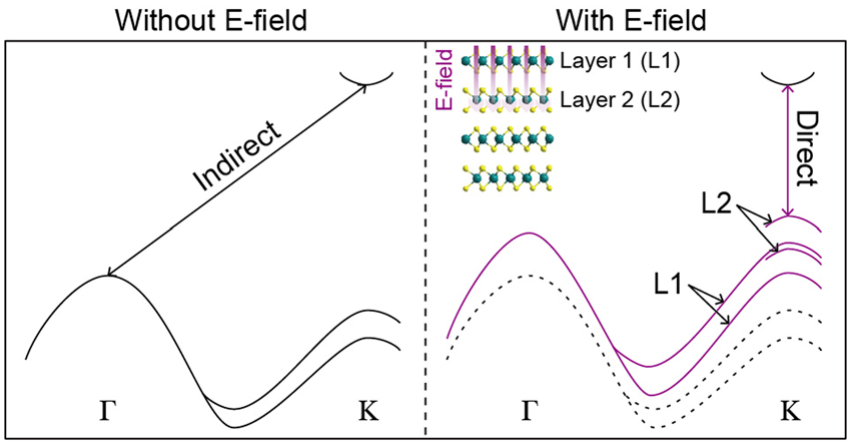
Schematic of electronic structure change in MoSe_2_ upon application of an electric field. The band structure of MoSe_2_ along the Γ-K direction without (left) and with (right) an external electric field applied perpendicular to the layers. The nature of the gap is also marked by the arrows. The inset illustrates the crystal structure and an applied electric field. L1 and L2 refer to the surface and sub-surface layers, respectively.

Figure 1 illustrates how the band structure of MX_2_ evolves as an electric field is applied in the calculation results. The band gap is generally reduced but faster at the K-point compared to the Γ-point. Eventually, MX_2_ will have a direct band gap at the K-point when the field becomes strong enough. Note that only the bands near the K-point are clearly split by the electric field due to the localized character of the wave function within each layer. Electronic states of the lower bands (L2) and upper bands (L1) mainly originate from the surface and sub-surface layers, respectively.

A possible way to induce such a strong electric field on the surface layers of MX_2_ is by dosing alkali metal on the sample^4, 32, 37, 38^. The electric field is induced by the ionized alkali metal layer when electrons are donated to the sample, and is expected to exist only within the few surface layers of MX_2_. The electric field strength can be effectively controlled by controlling the alkali metal dosing amount. More importantly, surface sensitive APRES technique can be used to measure the band dispersions.

### Electronic structure evolution of MoSe_2_ by dosing Rb on the surface

**Figure 2:**
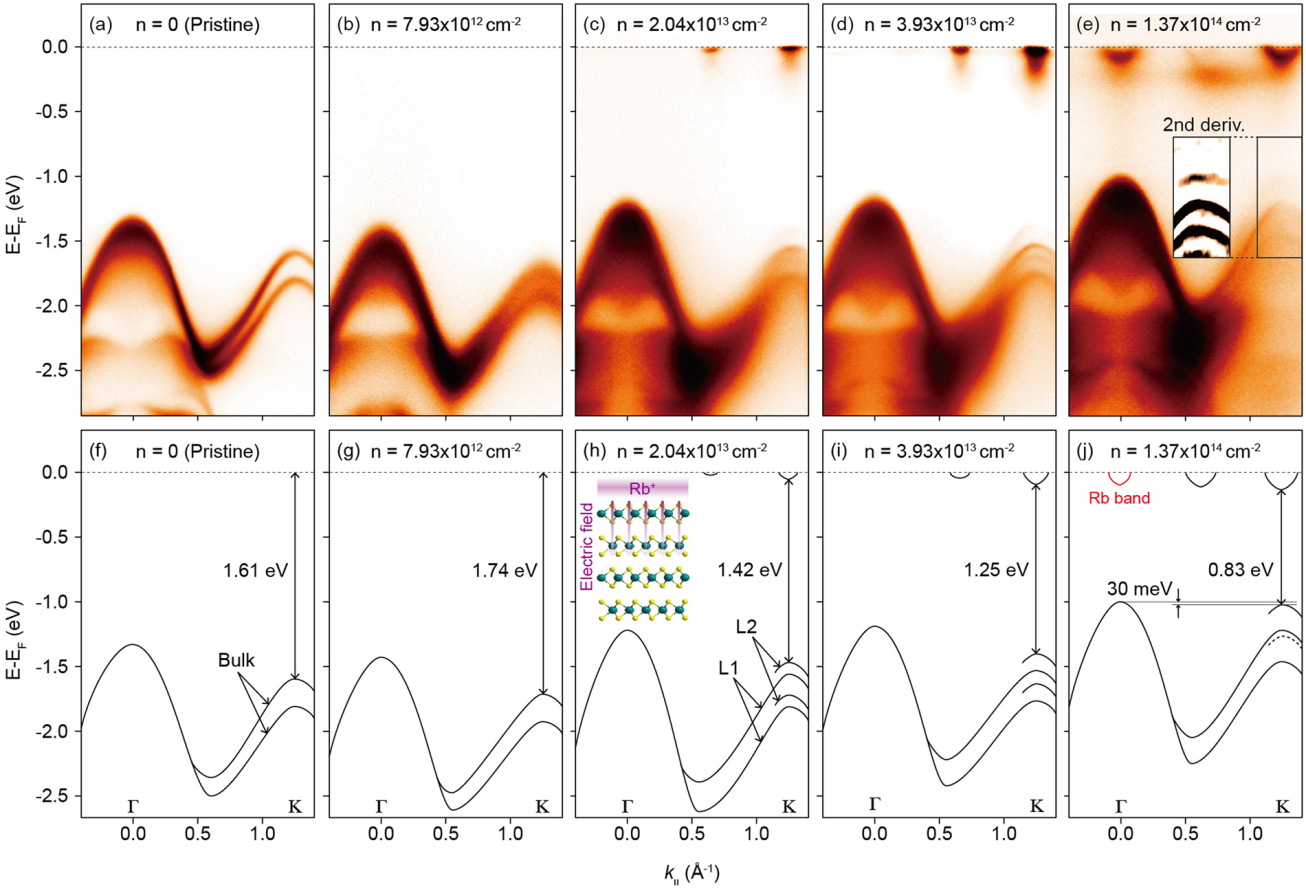
Rb dosing dependent electronic structure of MoSe_2_. (**a**–**e**) Γ-K ARPES data as a function of Rb dosing. *n* indicates the doped electron density as a result of Rb dosing. The inset in panel (**e**) is the second derivative of the boxed K-point data. Shown at the top of the panels are estimated electron densities. (**f**–**j**) The band structure determined from the ARPES data. The inset in panel (**h**) shows a schematic figure for an electric field in Rb dosed MoSe_2_. The red solid curve near the Fermi energy in (**j**) denotes the Rb band. The black dashed line in the valence band is the deduced lower L2 band which is not clearly distinguished in the data.

In our experiment, Rb is used for dosing. Figure 2 shows the ARPES data taken along the Γ-K direction for various Rb amounts and dispersions determined from the data. Figure 2(a) shows ARPES data from pristine MoSe_2_. We can clearly see the valence band structure which is consistent with published results^4^. As the Fermi level (*E*_*F*_) is in the middle of the gap, we only see the valence band. With a small amount of Rb dosing, electrons are also doped into the sample and *E*_*F*_ shifts to the CBM, causing downward shift of the valence band as seen in Fig. 2(b), compared to the data from the pristine sample in Fig. 2(a). In principle, the very bottom of the conduction band should be visible but the intensity is too weak to be observed.

As the dosing amount increases further, more electrons are doped into the sample and bottom of the conduction band appears at the K-point as well as ∑-point (located half way between Γ- and K-points) as seen in Fig. 2(c). The doped electron density (*n*) can be measured from the Fermi surface volume of the conduction bands and is estimated to be about 2 × 10^13^ cm^−2^. Data taken with even more Rb dosing show further downward shift of the conduction band and upward shift for the valence band as plotted in Fig. 2(d) and (e). Here, we note that after formation of Rb monolayer, electron transfer from Rb atom to the system does not occur for the additional Rb dosing on top of Rb monolayer. Therefore, there is a limit in the electron doping concentration using this method. The maximum electron doping we could obtain from the dosing experiment was about *n*_*max*_ ≈ 1.4 × 10^14^ cm^−2^ which also sets the maximum induced electric field through the dosing method. The band near *E*_*F*_ at the Γ-point for high *n* data is the free electron-like Rb band. In order to follow the band dispersion near the K-point, second derivative data is plotted as an inset in Fig. 2(e). Figure 2(f)–(j) plot the band dispersions along the Γ-K direction extracted from the ARPES data in Fig. 2(a)–(e).

There are a few notable aspects of the data. The intensity of the conduction band at the K-point is much stronger compared to that at the ∑-point, indicating that the CBM is located at the K-point. As for the valence band, the splitting of the bands to L1 and L2 bands near the K-point (clearly seen in Fig. 2(d)) occurs due to the surface electric field as previously discussed. Overall, the valence band shows an upward shift as seen in Fig. 2(c)–(e) with the doping increase, contrary to what is expected from electron doping. We attribute this behavior to decrease in the band gap size due to the screening effect from the doped carriers^39^. The most important aspect of the dosing dependent band evolution is that the VBM at the K-point rises faster than that at the Γ-point when the surface electron doping increases. As a result, binding energies of the VBM at Γ and K for the maximum electron doping shown in Fig. 2(e) and (j) are almost the same. That is, the system is on the verge of indirect to direct band-gap transition. Readers are reminded that electron doping is directly related to the strength of the electric field.

**Figure 3:**
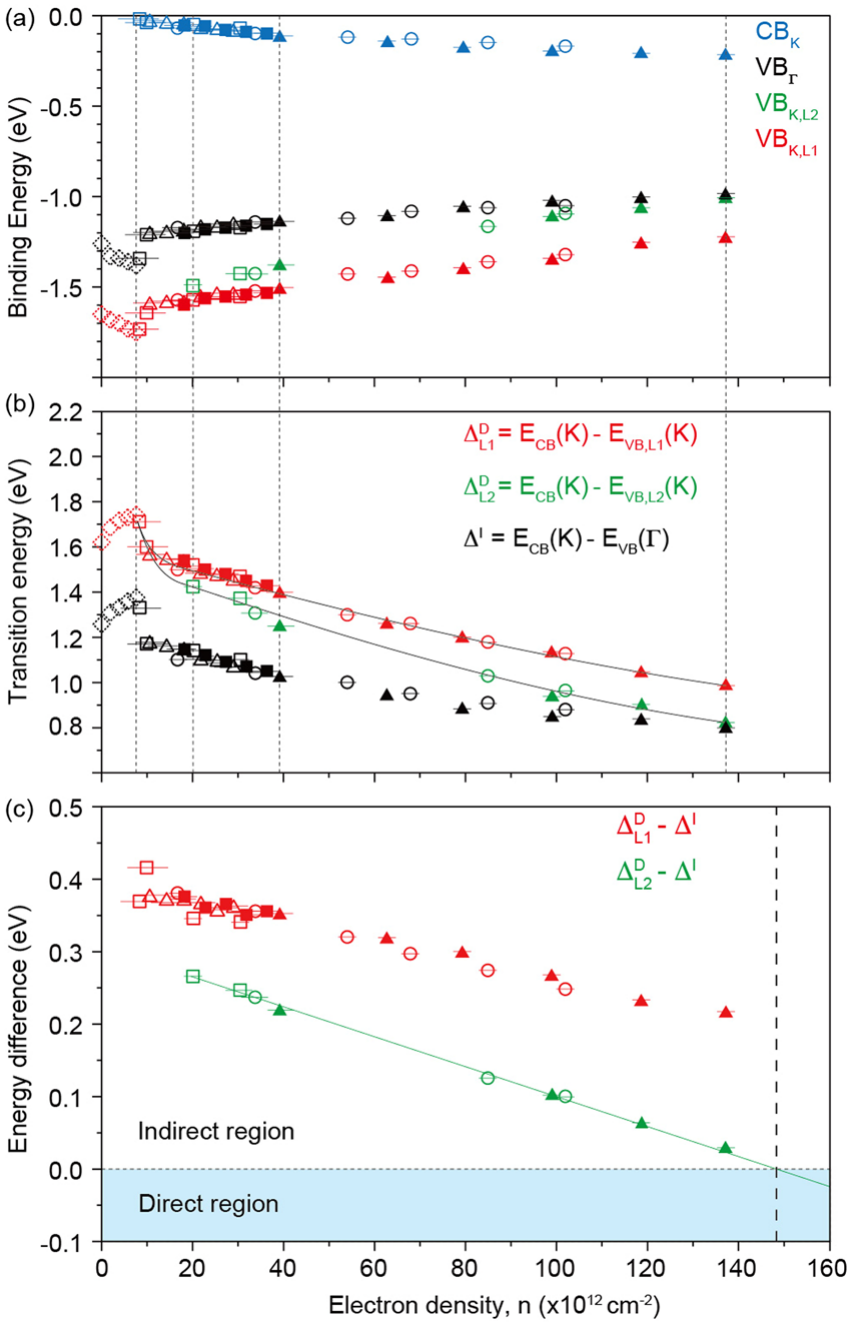
Dosing dependent evolution of the band gap. (**a**) Binding energies of VB_Γ_, VB_K,L1_ and VB_K,L2_ as a function of surface doping concentration. CB_K_ is also shown. Data points drawn with different symbols come from different data sets. Dotted symbols in the low doping region indicate the data from the doping concentration in which CBM could not be observed. Vertical black dotted lines mark the electron densities for the data presented in Fig. 2(b)–(e). (**b**) Doping dependence of the direct gaps ${{\rm{\Delta }}}_{L1}^{D}$ = CB_K_ − VB_K,L1_ and ${{\rm{\Delta }}}_{L2}^{D}$ = CB_K_ − VB_K,L2_, and indirect gap Δ^*I*^ = CB_K_ − VB_Γ_. In the low doping region where CBM is not seen, the gap size is referenced to the Fermi energy. (**c**) The differences between the indirect band gap and two direct band gaps at the K-point. A negative value for ${{\rm{\Delta }}}_{L2}^{D}$ − Δ^*I*^ indicates transition to a direct gap.

Focusing on the gap behavior, we plot in Fig. 3(a) the energy positions of VBM at Γ (VB_Γ_), L1 and L2 at K (VB_K,L1_ and VB_K,L2_, respectively) and CBM (CB_K_) as a function of the electron density. Vertical dotted lines indicate the electron densities for which ARPES data are shown in Fig. 2. VBM position at both Γ and K descends but CBM is not seen yet up to electron density of 8 × 10^12^ cm^−2^. Therefore, the gap size cannot be determined and the symbols show the energies relative to the Fermi energy. Starting from *n* ≈ 8 × 10^12^ cm^−2^, local VBMs at Γ and K begin to ascend while CBM becomes visible and descends as the conduction band is filled. We notice that VB_K,L2_ moves faster than VB_Γ_ and almost catches up with VB_Γ_ at *n*_*max*_.

More useful information can be obtained by calculating the energy differences among CB_K_, VB_K,L1_, VB_K,L2_, and VB_Γ_ as shown in Fig. 3(b). Note again that we cannot measure the energy in reference to CBM for n lower than 8 × 10^12^ cm^−2^. The energy differences Δ^*I*^, ${{\rm{\Delta }}}_{L1}^{D}$ and ${{\rm{\Delta }}}_{L2}^{D}$ (see the caption for the definition) show an initial abrupt decrease right after n ≈ 8 × 10^12^ cm^−2^ but begin to gradually decrease with more doping. Such gap behavior in the doping dependence originates from enhancement of screening effect by doped electrons, which is consistent with recent theoretical calculation results^39^.

The energy difference between CB_K_ and VB_Γ_ is smaller than that of CB_K_ and VB_K,L2_ meaning an indirect band gap semiconductor. However, values for the two are getting closer as *n* approaches *n*_*max*_. The differences between the direct band gap and indirect band gap sizes as a function of electron doping concentration are plotted in Fig. 3(c). Remarkably, the difference continually decreases with more doping and reveals a fairly linear behavior as shown in Fig. 3(c). This strongly suggest that a stronger electric field which corresponds to 1.5 × 10^14^ cm^−2^ can induce an indirect to direct band gap transition, as indicated by the vertical dashed line in the figure.

We note a similar work by another group published after we finished our work^40^. They performed ARPES on MoS_2_, MoSe_2_, MoTe_2_, WS_2_ and WSe_2_ with Rb dosing and found that only MoTe_2_ showed the indirect to direct band gap transition. The possible reason that MoSe_2_ did not show the transition was the insufficient surface doping concentration used in their experiment; we used three times higher surface doping concentration in MoSe_2_ than in their work and subsequently could reach the point very close to the band gap transition. Overall, our results here are consistent with their work and show that the band gap transition is a general feature of MX_2_ systems.

## Discussion

Rb dosing on the surface has two main effects on the system: surface electron doping and surface electric field. In general, increase in the electron doping enhances screening and reduces the band gap^39^. In fact, it has been already experimentally shown that the gap size of MX_2_ thin films can be tuned by controlling the screening effect, for example, by using different substrates^4^. Our experimental results in Fig. 3(b) provides systematic information on this issue. However, it is not intuitively easy to understand that the enhanced screening effect can affect the electronic structure non-uniformly in the momentum space and induce indirect to direct band gap transition. On the other hand, such transition can be attributed to the Stark effect from the surface electric field. The Stark effect can affect electronic states in a momentum dependent way since electronic states can have strong momentum dependence in the orbital character even within the same band. The valence band of MX_2_ indeed has momentum dependent orbital character. Not only multilayer but also ML MX_2_ show that valence band at K rises faster than the valence band at Γ^23, 26^. The strong Stark effect observed in the semiconducting black phosphorus is similar to what we observed^41^. Therefore, our work proposes a new way to induce direct band gap semiconductor in multilayer MX_2_, that is, by applying an electric field. This should be of significant importance for optoelectronics applications of MX_2_.

## Methods

### ARPES measurement

ARPES measurements were performed at the Beam line (BL) 4.0.3 of the Advanced Light Source, Lawrence Berkeley National Laboratory. Surface electron doping was carried out by Rb evaporation on the sample surface using commercial SAES Getters alkali metal dispensers. ARPES spectra were taken with Scienta R8000 electron analyzers. Data were taken with 58 eV photon energy and total energy resolution was better than 20 meV. Samples were cleaved and surface Rb dosing was done at a temperature below 50 K in an ultra-high vacuum better than 5 × 10^−11^ Torr. All measurements were performed within three hours after cleaving the sample.

### Data analysis

The surface electron doping concentration is estimated by calculating the Luttinger area of the conduction band Fermi surfaces at ∑ and K, as described in ref. 37. Electrons in the Rb band were not counted. Valley multiplicity of the ∑- and K-points are 6 and 2, respectively. In order to calculate the Fermi surface volume, Fermi momentum is determined by fitting the momentum distribution curves at the Fermi energy with a Lorentzian function. For the system in which doping concentration is too low to observe Fermi surfaces clearly, we assume that the electron doping concentration is proportional to Rb evaporation time. Error bars for electron doping concentrations in Fig. 3 are calculated from the fitting error bars in determining the Fermi momentum. The energy of the CBM at K and the VBM at Γ point is determined from the onset of the photoemission intensity. On the other hand, we use the peak position of the spectrum to determine the energy of the L1 and L2 at the K-point. Error bars for the energy positions in Fig. 3 are smaller than symbols.
